# Lattice-tip vs. standard irrigated focal-tip catheter for radiofrequency ablation of the cavotricuspid isthmus - the LINEAR randomized trial

**DOI:** 10.1093/europace/euag046

**Published:** 2026-04-14

**Authors:** Stylianos Tzeis, Dimitrios Asvestas, Vasileios Sousonis, Emmanouil Vavouris, Paschalis Karakasis, Dimitrios Charitos, Stavros Karanikas, Konstantinos Pamporis, Panos Vardas, Konstantinos Vlachos

**Affiliations:** Department of Cardiology, Mitera Hospital, 6 Erythtrou Stavrou str, 151 23, Marousi, Athens, Greece; Department of Cardiology, Mitera Hospital, 6 Erythtrou Stavrou str, 151 23, Marousi, Athens, Greece; Department of Cardiology, Mitera Hospital, 6 Erythtrou Stavrou str, 151 23, Marousi, Athens, Greece; Department of Cardiology, Mitera Hospital, 6 Erythtrou Stavrou str, 151 23, Marousi, Athens, Greece; 7th Cardiology Department, Hygeia Hospital, Athens, Greece; Department of Cardiology, Mitera Hospital, 6 Erythtrou Stavrou str, 151 23, Marousi, Athens, Greece; Department of Cardiology, Mitera Hospital, 6 Erythtrou Stavrou str, 151 23, Marousi, Athens, Greece; 7th Cardiology Department, Hygeia Hospital, Athens, Greece; 7th Cardiology Department, Hygeia Hospital, Athens, Greece; 7th Cardiology Department, Hygeia Hospital, Athens, Greece

**Keywords:** Atrial flutter, Cavotricuspid isthmus, Catheter ablation, Lattice-tip catheter, Irrigated-tip catheter

## Abstract

**Aims:**

Cavotricuspid isthmus (CTI) ablation is a cornerstone therapy for typical atrial flutter (AFl) and is commonly performed during atrial fibrillation (AF) ablation. In this multicentre randomized trial, we compared a lattice-tip catheter with an irrigated focal-tip catheter for radiofrequency CTI ablation (LINEAR study—ClinicalTrials.gov NCT07078760).

**Methods and results:**

Patients were randomized to a lattice-tip, dual-energy catheter (lattice-tip group) or to a standard 3.5-mm irrigated radiofrequency catheter (standard group) in two centres. In the lattice-tip group, only radiofrequency was utilized. The primary endpoint was the achievement and persistence of bidirectional CTI block after a 60-minute waiting period, confirmed by high-density electroanatomical mapping and adenosine testing. Secondary endpoints included the rate of first-pass block, the number of lesions, and the ablation time. Procedural complications were recorded. In total, 102 patients were randomized. The primary endpoint was achieved in significantly more patients in the lattice-tip as compared to the standard group (94.1% vs. 68.6%, *P* = 0.002). The lattice-tip catheter resulted in a significantly higher rate of first-pass block (90.2% vs. 60.8%, *P* = 0.001). CTI block required significantly shorter ablation time (41.3 ± 12.1 vs. 245.3 ± 91.3 s, *P* < 0.001) and a significantly lower number of lesions (8.3 ± 2.4 vs. 13.4 ± 4.5, *P* < 0.001) in the lattice-tip as compared to the standard group. No procedural complications were documented.

**Conclusion:**

The lattice tip catheter resulted in higher acute procedural success for radiofrequency CTI ablation compared to the standard irrigated focal-tip catheter. Future studies are needed to assess long-term efficacy and clinical outcomes.

## Graphical Abstract

**Graphical Abstract:**
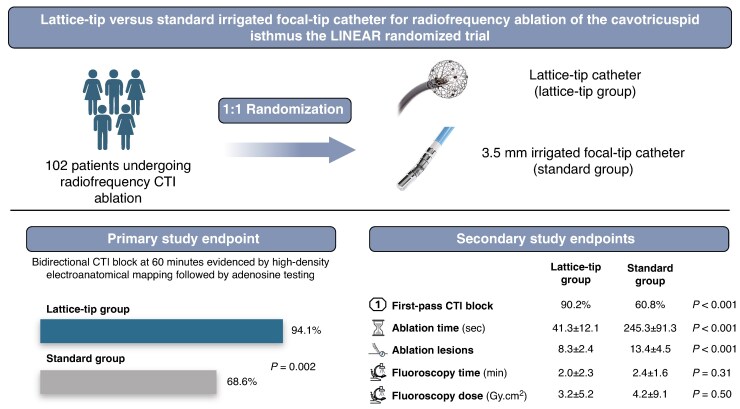

What’s New

- The lattice-tip catheter demonstrated superior efficacy compared with the standard irrigated focal-tip catheter in achieving acute bidirectional block following radiofrequency ablation of the cavotricuspid isthmus (CTI).
- The lattice-tip catheter was associated with higher procedural efficiency as evidenced by a significant reduction in ablation time, number of lesions, and procedural time to achieve CTI block.

## Introduction

Catheter ablation is the recommended treatment for patients with cavotricuspid isthmus (CTI)—dependent atrial flutter (AFl) due to its enhanced efficacy combined with low risk of procedural complications. Based on a recent international clinical consensus, catheter ablation is recommended for recurrent symptomatic peritricuspid macroreentrant atrial tachycardias and should also be considered even after the first episode (except when occurring within 30 days after surgery) regardless of patient’s age.^1^ Cavotricuspid isthmus ablation is also performed as part of adjunctive lesion sets beyond pulmonary vein isolation (PVI) in patients with persistent atrial fibrillation (AF).^2–4^

Radiofrequency energy remains the standard energy source for CTI ablation due to its proven efficacy and favourable safety. The 2025 EHRA/APHRS/LAHRS/AEPC clinical consensus statement advises the use of single-tip irrigated catheters, preferably with contact force, as the optimal choice for radiofrequency ablation of atrial tachycardias.^1^ The use of pulsed field ablation (PFA) for CTI is limited by the risk of coronary artery vasospasm, which remains a clinical concern even after the implementation of preventive measures.^5–7^

The procedural endpoint of CTI ablation is the achievement of bidirectional block across the ablation line.^1,4^ Validation of bidirectional block has been historically performed with differential pacing manoeuvres.^8^ However, recent data cast doubt on the accuracy of this validation method due to a non-negligible rate of false-positive diagnosis of bidirectional block due to the presence of endocardial residual slow conduction or epicardial bridging across the lines.^9^ The use of high-resolution mapping has been shown to overcome this caveat and unmask the presence of pseudoblocks that may otherwise be falsely diagnosed using conventional differential pacing.^9^

The introduction of new catheter systems has provided additive options for catheter ablation.^10^ A novel 7.5 F large footprint irrigated catheter with an expandable 9 mm spheroid-shaped lattice electrode tip capable of toggling between radiofrequency and PFA energy has been used for AFl and AF ablation.^11–13^ However, a direct comparison of the procedural performance between the lattice-tip catheter and the standard irrigated focal-tip catheter for CTI ablation is currently lacking. In this investigator-initiated, prospective, multicentre, randomized trial (the LINEAR study), we compared the lattice-tip vs. a standard irrigated focal-tip catheter in consecutive patients scheduled for CTI ablation, either as a standalone procedure for CTI-dependent AFl or as part of an AF ablation workflow, using high-resolution mapping for validation of bidirectional block.

## Methods

### Study design

The Lattice-tip vs. Standard Irrigated Focal-tip Catheter for Linear Ablation of the Cavotricuspid Isthmus (LINEAR) trial was an investigator-initiated, prospective, open-label, superiority randomized clinical trial that was conducted in 2 centres in Greece. The study was approved by the ethics committee at each site and conformed to the ethical guidelines of the Declaration of Helsinki. All participating patients provided written informed consent. The conduct of this trial was based on a pre-specified protocol in ClinicalTrials.gov (NCT07078760), which was followed without modifications.

### Study population

Consecutive patients with CTI-dependent AFl and/or AF scheduled for CTI ablation, either as a standalone procedure or as part of an AF ablation workflow, were considered eligible for participation in the trial. Prior CTI ablation was considered an exclusion criterion. All eligible patients were randomized either to a lattice-tip, dual-energy catheter (Sphere-9™, Medtronic, lattice-tip group) or to a standard 3.5 mm irrigated-tip catheter (ThermoCool® SmartTouch SF™, J&J MedTech, standard group).

### Ablation procedure

All participants received anticoagulation therapy for a minimum of one month before the ablation procedure. Catheter ablation was performed under uninterrupted or minimally interrupted (skipped morning dose of twice-daily administered direct oral anticoagulants) anticoagulation therapy. Antiarrhythmic medications, except amiodarone, were discontinued for at least five half-lives before the procedure. Amiodarone was discontinued one month before the scheduled ablation. General anaesthesia was utilized in all patients. Venous access was obtained via the right femoral vein under echocardiographic guidance in all patients. Twelve-lead electrocardiogram and intracardiac electrograms were recorded and analysed using the LABSYSTEM^TM^ PRO EP Recording System (Boston Scientific).

A steerable decapolar electrode catheter was positioned in the coronary sinus (CS). In patients presenting in sinus rhythm, ablation was performed during pacing from the proximal CS bipole at a cycle length of 600 ms. In patients presenting with ongoing tachycardia, CTI-dependent flutter was confirmed using activation mapping combined with standard entrainment manoeuvres.

CTI ablation was performed using a point-by-point technique, creating a contiguous line of lesions along the CTI, guided by fluoroscopy and electroanatomical mapping. Lesions were delivered exclusively using radiofrequency energy in both groups. The catheter was gradually dragged from the tricuspid valve annulus towards the inferior vena cava after completion of each lesion. In the lattice-tip group, the saline irrigation rate was 4 mL/min during mapping and 30 mL/min during ablation. Radiofrequency energy was delivered using recommended settings (5 s per lesion, level 80%) with a target temperature of 73°C. PFA was not used for CTI ablation in any patient in the lattice-tip group. In the standard group, the power setting was 30 W with a target contact force of 10–30 g and a target ablation index (AI) value of 450. Saline irrigation was set to 2 mL/min during mapping and increased to 8 mL/min during ablation. The temperature limit was set to 43°C. The VISITAG module was utilized to tag the location of each lesion, with the following settings: Stability range of 2–3 mm, stability duration of 3–5 s, force over time of 25%, and a tag size diameter of 3 mm.

Initial achievement of bidirectional CTI block was assessed using standard differential pacing manoeuvres from the CS ostium and the right atrial lateral wall, supplemented by high-density electroanatomical mapping with emphasis on proximity to the line to exclude the presence of endocardial slow conduction and epicardial connections.^9^ Activation mapping was performed using either the Affera (Medtronic) mapping system combined with the lattice-tip catheter (lattice-tip group) or the CARTO mapping system (J&J MedTech) and a multipolar catheter (PENTARAY or OCTARAY, J&J MedTech, standard group). First-pass CTI block was defined as the achievement of a bidirectional CTI block upon the completion of the initially deployed CTI line, without the need for additional lesions. CTI length was defined as the distance between the most anterior (tricuspid valve) and the most posterior deployed lesion (inferior vena cava).

In patients undergoing adjunctive AF ablation, PVI was achieved with the respective ablation catheters. In the lattice-tip group, the deployment of PFA lesions was allowed for left atrial (LA) lesions at the discretion of the physician.

After a 60-minute waiting period following the initial documentation of a bidirectional CTI block, the ablation line was reassessed for spontaneous recovery of conduction, using differential pacing criteria and high-density electroanatomical mapping. In the absence of spontaneous recovery, adenosine testing was performed to assess the presence of dormant CTI conduction. An initial adenosine dose of 0.1 mg/kg was rapidly infused intravenously, aiming to achieve at least one blocked *P* wave. In case of inability to achieve transient atrioventricular block, the infused dose of adenosine was increased by increments of 3 mg. Testing was initially performed under pacing from the proximal coronary sinus, with the multipolar catheter positioned just laterally to the ablation line, and repeated during pacing from the mapping catheter just laterally to the CTI ablation line. Dormant recovery of conduction was defined as transient or permanent shortening of the transisthmus conduction interval, following adenosine infusion.

The following procedural parameters were systematically recorded: transisthmus conduction interval from the proximal coronary sinus to the lateral isthmus and vice versa following CTI block, time from entry to the right atrium to bidirectional CTI block, total number of CTI lesions, total ablation time, and fluoroscopy time and dose to achieve initial bidirectional CTI block.

### Study endpoints

The primary study endpoint was the achievement and persistence of bidirectional CTI block after a 60-minute waiting period, as evidenced by standard differential pacing manoeuvres and high-density electroanatomical mapping followed by adenosine testing.

Secondary study endpoints included the rate of first-pass CTI block, the total number of ablation lesions, the total ablation time, the time from entry to the right atrium to bidirectional CTI block, and the fluoroscopy time and dose from vascular access to achievement of bidirectional CTI block.

Procedural complications were systematically recorded.

### Statistical analysis

Based on the study by Vlachos et al., in which 38% of CTI lines demonstrated residual conduction on high-density mapping,^9^ we anticipated that approximately 60% of patients in the standard group would have persistent bidirectional CTI block assessed by high-density electroanatomical mapping at 60 min. Based on preliminary studies conducted by our group, the corresponding proportion in the lattice-tip group was expected to be 85% (absolute difference: 25% points). Under a superiority framework with two-sided α = 0.05, 1:1 allocation, and 80% power, the required sample size was estimated at 51 patients per group (102 total) to detect the anticipated between-group difference in proportions. As the primary endpoint was assessed intra-procedurally, dropouts were not anticipated; therefore, no further inflation or adjustment to the target sample size was applied.

Patients were randomized in a 1:1 allocation to the compared groups. Randomization was centrally implemented via a secure, interactive web-based response system to preserve allocation concealment. The assignment sequence was computer-generated using randomly permuted blocks and was stratified by trial centre to ensure balanced treatment distribution across sites. Investigators did not have access to the randomization sequence throughout the study period.

Categorical variables are summarized as counts and percentages, and continuous variables are described as mean ± SD when normally distributed. Distributional assumptions were evaluated using histograms and Q–Q plots, complemented by the Shapiro–Wilk test. Given the known sensitivity of formal normality testing to sample size, decisions regarding parametric inference were driven primarily by graphical diagnostics, the presence of skewness/outliers, and the plausibility of approximate normality at the group level.

Between-group comparisons for continuous outcomes were performed using Student’s *t*-test under the assumptions of independent observations and approximate normality of the underlying outcome within each treatment group. Homoscedasticity was assessed by comparing group variances; when evidence of unequal variances was present, the Welch *t*-test was applied to preserve nominal type I error.

For categorical outcomes, group differences were assessed using Pearson’s χ^2^ test when the asymptotic approximation was justified. In the presence of sparse data—defined *a priori* as any expected cell count <5—Fisher’s exact test was used to provide valid exact inference.

Missing data were handled using a complete-case analysis approach. This strategy was considered appropriate because missingness was minimal (<5%) and restricted to secondary variables, limiting the potential for meaningful bias or loss of efficiency in primary comparisons. No imputation was undertaken.

All statistical tests were two-sided, and statistical significance was set at *P* < 0.05. All analyses were performed with R statistical software (v. 4.5.1).

## Results

### Baseline characteristics

In total 102 patients participated in the trial (mean age 64.4 ± 10.6 years, 66% males, 53.9% hypertension, mean CHADSVA score 1.62 ± 1.26). Baseline characteristics are presented in detail in *Table 1*. There was no significant difference in any of the baseline characteristics between the compared groups (Τable 1).

**Table 1:** Baseline characteristics

Baseline characteristics	Overall *n* = 102	Lattice-tip group *n* = 51	Standard group *n* = 51	*P*-value
Female gender	35 (34.3%)	17 (33.3%)	18 (35.3%)	0.83
Age (years)	64.4 ± 10.6	63.7 ± 10.9	65.1 ± 10.4	0.52
BMI (kg/m^2^)	27.9 ± 4.3	28.5 ± 4.6	27.3 ± 3.9	0.18
History of AF	93 (91.2%)	48 (94.1%)	45 (88.2%)	0.49
CHF	13 (12.8%)	9 (17.7%)	4 (7.8%)	0.14
PAD	2 (2.0%)	1 (2.0%)	1 (2.0%)	>0.99
CAD	14 (13.7%)	6 (11.8%)	8 (15.7%)	0.56
Hypertension	55 (53.9%)	28 (54.9%)	27 (52.9%)	0.84
Diabetes	9 (8.8%)	6 (11.8%)	3 (5.9%)	0.49
Stroke/TIA	0 (0%)	0 (0%)	0 (0%)	>0.99
CHADSVA score	1.62 ± 1.26	1.67 ± 1.34	1.59 ± 1.19	0.75

Data are presented as *n* (%) or as mean ± standard deviation.

AF, atrial fibrillation; BMI, body mass index; CAD, coronary artery disease; CHF, congestive heart failure; PAD, peripheral arterial disease; TIA, transient ischaemic attack

### Procedural characteristics

Among the enrolled patients, 8 underwent only CTI ablation for AFl, while 94 had CTI ablation as part of an AF ablation workflow (16 patients with documented preprocedural AFl, and 78 patients as part of an extensive lesion set for persistent AF). Procedural characteristics are reported in detail in *Table 2*. CTI length did not differ significantly between the lattice-tip and the standard group (32.1 ± 7.0 mm vs. 29.6 ± 7.3 mm, respectively, *P* = 0.09).

**Table 2:** Procedural characteristics

Procedural characteristic	Lattice-tip group	Standard group	*P*-value
Procedure type			
AFl ablation only	2 (3.9)	6 (11.8)	0.27
AFl and AF ablation	49 (96.1)	45 (88.2)	
Total ablation time (sec)	41.3 ± 12.1	245.3 ± 91.3	<0.001
Total number of ablation lesions	8.3 ± 2.4	13.4 ± 4.5	<0.001
Time from RA entry to CTI block (min)	10.5 ± 7.0	21.6 ± 12.4	<0.001
Fluoroscopy time to achieve CTI block (min)	2.0 ± 2.3	2.4 ± 1.6	0.31
Fluoroscopy dose to achieve CTI block (Gy.cm^2^)	3.2 ± 5.2	4.2 ± 9.1	0.50
Effective adenosine dose (mg)	13.7	13.5	0.73
Postablation transisthmus interval (proximal CS to lateral isthmus, ms)	169.9 ± 24.5	162.9 ± 34.0	0.24
Postablation transisthmus interval (lateral isthmus to proximal CS, ms)	167.2 ± 25.4	163.6 ± 34.0	0.32

Data are presented as *n* (%) or as mean ± standard deviation.

AF, atrial fibrillation; AFl, atrial flutter; CS, coronary sinus; CTI, cavotricuspid isthmus; RA, right atrium

### Outcome measures

**Figure 1:**
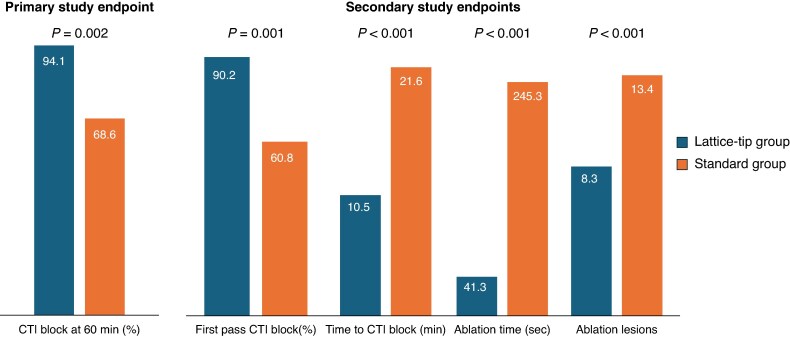
Graphical presentation of the primary and secondary study endpoints. CTI: cavotricuspid isthmus.

A significantly higher percentage of patients in the lattice-tip group reached the primary endpoint as compared to the standard group (94.1% vs. 68.6% respectively, *P* = 0.002, *Figure 1*). In the lattice-tip group, 3 of 51 patients failed to reach the primary endpoint: 2 exhibited spontaneous recovery and 1 patient showed adenosine-mediated recovery of CTI conduction after the 60-minute waiting period. Additional radiofrequency lesions were delivered at sites of conduction gaps in the 3 lattice-tip group patients with CTI conduction recovery, resulting in bidirectional CTI block. In the standard group, 16 patients did not reach the primary endpoint, including 4 with failure to achieve bidirectional CTI block, 11 with spontaneous and 1 with transient adenosine-mediated recovery of CTI conduction following completion of the waiting period. In the 11 patients with spontaneous recovery of CTI conduction, bidirectional block was subsequently achieved with additional lesions targeting sites of conduction gaps. No further lesions were delivered at the end of the waiting period in the 4 patients who failed to achieve initial CTI block. Mapping of residual conduction was not possible in the patient with transient, adenosine-mediated recovery of CTI conduction.

**Figure 2:**
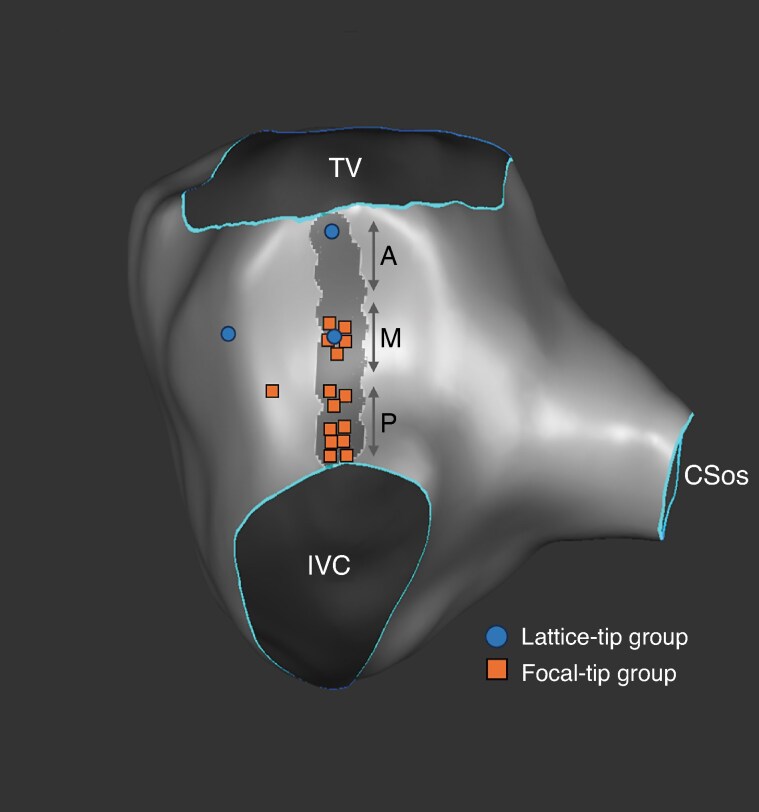
Distribution of residual conduction sites across the CTI in patients who did not reach the primary study endpoint. The dark grey zone represents the ablation line. Residual conduction sites are indicated by blue circles (lattice-tip group) or orange squares (standard group). The anterior, mid and posterior third of the CTI are designated as A, M and P, respectively. CTI: cavotricuspid isthmus, CSos: coronary sinus ostium, IVC: inferior vena cava, TV: tricuspid valve.

**Figure 3:**
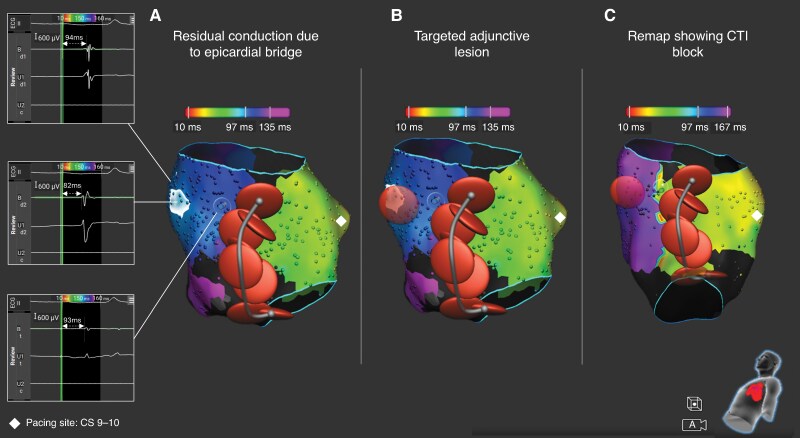
Residual conduction across the CTI due to epicardial bridging in a patient from the lattice-tip group. After deployment of a contiguous line of lesions across the CTI, high-density activation mapping during pacing from the proximal bipole of the coronary sinus catheter showed epicardial conduction bridging (*A*). Delivery of an additional lesion at this site (*B*) resulted in CTI conduction block as validated by a redo activation map (*C*). CTI: cavotricuspid isthmus.

**Figure 4:**
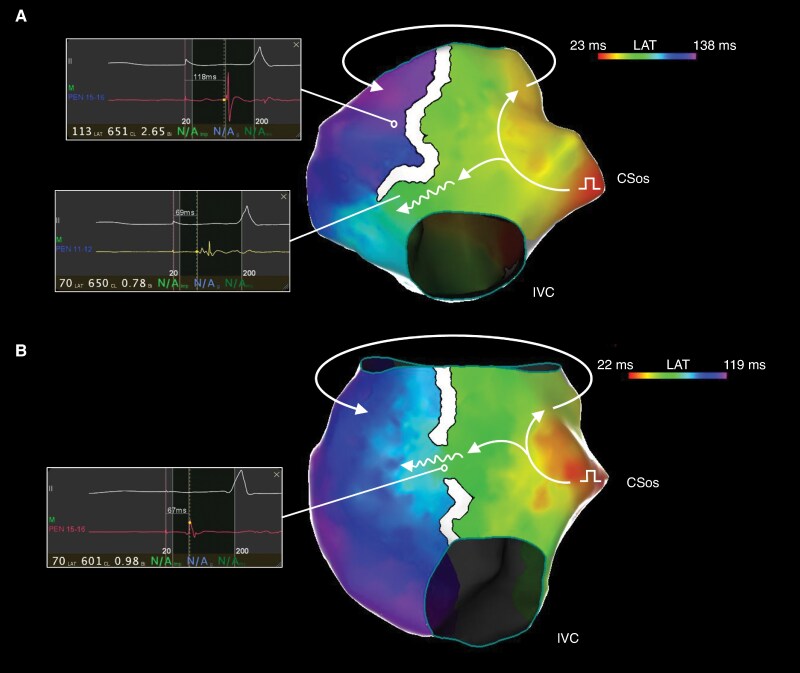
Residual conduction across the CTI in two patients from the standard group assessed with high-density activation mapping during pacing from the proximal bipole of the coronary sinus catheter. *(A*) Residual conduction in the posterior third of the CTI. *(B*) Residual conduction in the mid third of the CTI. CTI: cavotricuspid isthmus, CSos: coronary sinus ostium, IVC: inferior vena cava.

The distribution of residual conduction gaps across the CTI as assessed with high-density electroanatomical mapping in patients who failed to reach the primary endpoint is shown in *Figure 2*. In the lattice-tip group, 1 conduction gap was located in the anterior third and 1 in the mid third of the CTI line, while 1 patient exhibited residual conduction due to epicardial bridging (*Figure 3*). In the standard group, residual conduction was demonstrated in the mid third of the CTI line in 5 patients (31.3%), in the posterior third in 9 patients (56.3%), while 1 patient (6.3%) exhibited residual conduction due to epicardial bridging (*Figure 2*). Mapping of residual conduction was not possible in 1 patient in the standard group due to transient recovery of CTI conduction during adenosine testing. Representative cases of residual conduction across the CTI in the standard group are shown in *Figure 4*.

In the total patient population, first-pass CTI block was achieved in 75.5% of patients. Achievement of first-pass CTI block was associated with a significantly higher likelihood of reaching the primary study endpoint (89.6% vs. 56%, *P* = 0.001). Furthermore, the rate of first-pass CTI block was significantly higher in the lattice-tip than in the standard group (90.2% vs. 60.8%, *P* = 0.001).

Achievement of CTI block required significantly shorter ablation time (41.3 ± 12.1 vs. 245.3 ± 91.3 s, *P* < 0.001) and a significantly lower number of ablation lesions (8.3 ± 2.4 vs. 13.4 ± 4.5, *P* < 0.001) in the lattice-tip as compared to the standard group.

The time from entry to the right atrium to the achievement of bidirectional CTI block (onset of the 60-minute waiting period) was significantly shorter in the lattice-tip group compared to the standard group (10.5 ± 7.0 vs. 21.6 ± 12.4 min, respectively, *P* < 0.001).

The fluoroscopy time and dose from vascular access to bidirectional CTI block (onset of the 60-minute waiting period) were similar in the lattice-tip and the standard group (2.0 ± 2.3 vs. 2.4 ± 1.6 min, *P* = 0.31; and 3.2 ± 5.2 vs. 4.2 ± 9.1 Gy.cm^2^, *P* = 0.50, respectively).

No procedural complications occurred in either group.

## Discussion

The major findings of the LINEAR trial are the following: (a) the lattice-tip catheter is superior to the standard irrigated focal tip catheter for achieving acute bidirectional block following radiofrequency ablation of the CTI. The superior acute efficacy was documented using stringent validation criteria including high-density electroanatomical mapping after a prolonged waiting period of 60 min followed by adenosine testing and (b) the lattice-tip catheter results in higher procedural efficiency as evidenced by significant reductions in ablation time, number of lesions and procedural time to achieve CTI block.

In this randomized multicentre trial, we compared two different ablation catheters for radiofrequency ablation of the CTI, performed either as a standalone procedure or as part of an extensive AF ablation workflow. The standard group underwent CTI ablation using an irrigated 3.5 mm focal-tip contact-force radiofrequency catheter which is currently considered the standard-of-care. The 2025 EHRA/APHRA/LAHRS/AEPC consensus document for the management of patients with atrial arrhythmias advises with the highest strength of evidence the use of single-tip irrigated catheters, preferably with contact force sensing, for radiofrequency ablation of atrial arrhythmias.^1^ During these procedures, real-time assessment of lesion quality is performed by guiding energy delivery using quantitative metrics, such as the AI.^14^ In contrast to AF ablation, where target AI values are well-established, the optimal radiofrequency energy ‘dosing’ and standardized thresholds for lesion quality indices in CTI ablation remain largely undefined.^15^ In a retrospective analysis, the optimal cutoff values for achieving first-pass CTI ablation were an AI >420 for the anterior segment and an AI >376 for the posterior side.^16^ Furthermore, a multicentre non-randomized trial of 412 consecutive patients from 31 centres concluded that a target AI of 500 resulted in a first-pass CTI conduction block rate of 88.3%. While no major complications were reported, three audible ‘pops’ occurred during energy delivery.^17^ In a previous randomized trial, our group showed that CTI ablation with sequential spot lesion deployment using a higher target force-time integral (FTI) of 600 gram-seconds did not increase acute procedural efficacy as compared to the standard AF ablation FTI cutoff of 400 gram-seconds.^18^ In the current study, we used a target ablation index of 450 aiming to ensure transmurality without impairing procedural safety.

In the second group we evaluated a novel ablation catheter that combines temperature-controlled radiofrequency energy delivery with a large 9 mm spherical, irrigated, lattice tip. The large surface area of the tip allows the delivery of very high-power (up to 800 W) ablation lesions in very short duration (5 s), maintaining low current density to prevent steam pop occurrence. Central catheter irrigation ensures homogeneous cooling to avoid tissue overheating. In the first-in-human clinical trial, this lattice-tip catheter demonstrated feasibility and safety for point-by-point pulmonary vein isolation, CTI and LA linear ablation.^11^ In a cohort of 43 patients undergoing radiofrequency ablation of the CTI as part of left atrial AF ablation procedures, the authors also demonstrated high procedural efficacy and efficiency using the same lattice-tip catheter.^13^

## Validation of acute CTI block

The establishment of bidirectional block across any type of linear lesion is the sine qua non to prevent proarrhythmic sequelae.^1,4,9^ The assessment of linear block has been traditionally performed using differential pacing manoeuvres.^8^ However, several studies have highlighted the caveats of relying solely on differential pacing for documentation of linear lesion integrity due to the associated risk of pseudoblock related to residual endocardial slow conduction or epicardial bridging.^9,19,20^ Vlachos et al. reported that 37% of CTI lines with residual conduction, as validated by high resolution mapping, would have been erroneously detected as blocked (pseudoblock) if only differential pacing was used.^9^ The undetected residual conduction impacts patient rhythm outcomes and increases the risk of atrial tachycardia recurrence during follow-up.^9^ Therefore, the systematic use of high-resolution mapping is considered the most stringent and reliable method to document bidirectional block and uncover the presence of pseudoblocks. In our study, we systematically implemented high-density activation mapping to assess the integrity of all CTI lesions, thus maximizing the accuracy and precision of line assessment.

Furthermore, we also systematically implemented a 60-minute waiting period before assessing the procedural primary endpoint. The incorporation of a waiting period after CTI ablation enhances the identification and subsequent ablation of residual gaps, significantly reducing recurrent atrial flutter rates.^21^ Theoretically, the longer the observation period, the more likely the detection of reconduction due to reversible or non-transmural ‘weak links’ within the linear lesion. A time-dependent occurrence of early pulmonary vein reconduction is well-documented in AF ablation.^22,23^ Although most reconnections occur within 30 min, a significant proportion still emerges between 30 and 60 min, becoming negligible after one hour.^22^ Given that the optimal waiting period following CTI ablation has not been well-defined, we utilized a 60-minute cutoff to maximize the detection of acute reconnections. Lastly, to further enhance the stringency of our protocol for detecting acute CTI reconduction, we also performed adenosine testing following the 60-minute waiting period to unmask dormant conduction.^24–26^

## Outcome measures

Radiofrequency ablation with the lattice-tip catheter resulted in higher procedural efficacy compared to the irrigated focal-tip catheter. The superior efficacy of the lattice-tip catheter may be attributed to its design that enables delivery of up to ≈10-fold higher current output compared to the standard irrigated focal-tip catheter. This is feasible without compromising procedural safety due to the 10-fold larger catheter surface area (275 mm^2^ vs. 28 mm^2^) that enables delivery of high current output while maintaining a low current density, thereby reducing the risk of tissue overheating.^27^ In a swine model of linear atrial ablation, the lattice-tip catheter produced wider and more durable lesions with increased ablation line continuity compared to standard irrigated catheters.^28^ In addition, the conformability of the lattice-tip catheter combined with its large thermal footprint and spherical design may facilitate catheter-tissue contact in challenging anatomies, such as pouches or prominent Eustachian ridge, compared to focal tip catheters. In consistency, high-density mapping in our trial demonstrated a clustering of the residual gaps in the posterior CTI segment in the standard tip group. In this area, a prominent Eustachian ridge may impair catheter stability and prevent optimal catheter-tissue contact. Procedural use of intracardiac echocardiography might have improved catheter–tissue contact and lesion contiguity in the standard group in cases of challenging anatomies, potentially enhancing acute efficacy; however, intracardiac echocardiography was not used for CTI ablation in our study to reflect common clinical practice. Another potential explanation for the reduced efficacy of the focal-tip catheter is the requirement for a significantly higher number of ablation lesions, which increases the risk of oedema and impairs lesion transmurality.

One could argue that the significant difference in procedural efficacy might be attributed to an unexpectedly low rate of CTI block in the standard group. However, the CTI block achievement rate in our control group was 68.6%, which is comparable to the rate of 62.4% reported by Vlachos et al. in a study that also employed high resolution mapping for validation of line integrity.^9^ Several studies relying exclusively on differential pacing have reported higher rates of CTI block, probably due to rate overestimation by the erroneous inclusion of pseudoblocks. In our study, the systematic use of high-density mapping after a 60-minute waiting period may explain the lower-than-expected rate of CTI block in the standard group compared with everyday clinical practice where differential pacing manoeuvres alone are usually implemented for assessment of acute efficacy.

In consistency with the superior primary endpoint achievement, the lattice-tip group also demonstrated a significantly higher rate of first-pass CTI block. Several studies have shown that first-pass CTI block is a predictor of a durable isthmus ablation line.^17,18^

The lattice-tip catheter also resulted in superior procedural efficiency as evidenced by significant reduction in the number of lesions, ablation time and time to achieve CTI block. This is attributed to the large thermal footprint of the catheter, which covers approximately 20–25% of the CTI line length. Therefore, the number of lesions required to achieve a contiguous ablation line along the CTI isthmus is reduced, resulting in a more streamlined ablation procedure.

The present findings, while derived from CTI ablation, may be viewed as a proof-of-concept that the lattice-tip catheter can enhance lesion contiguity and acute efficacy in atrial linear ablation more broadly, potentially extending to other atrial linear lesion sets. However, extrapolation to left atrial applications beyond PVI should be made with caution. Larger specifically designed studies with long-term follow-up are warranted to define the optimal modality for left atrial linear lesion sets.

## Study limitations

Energy dosing in the standard group was selected based on available evidence to enhance procedural efficacy while maintaining safety. However, it remains unknown whether the use of higher energy settings or higher AI targets would have resulted in improved outcomes in the standard group, potentially attenuating the observed superiority of the lattice-tip catheter in acute efficacy. In addition, a substantial proportion of study participants underwent CTI ablation as an adjunctive lesion set for persistent AF, which may impair the external validity of our findings to patients with clinically documented CTI-dependent AFl alone. Furthermore, long-term follow-up was not available, precluding conclusions regarding lesion durability and arrhythmia recurrence beyond the acute procedural endpoint. Despite the use of stringent surrogates for ablation durability in this trial, long-term follow-up studies are needed to demonstrate the clinical superiority of the lattice-tip catheter regarding patient rhythm outcome. Finally, although no procedural adverse events were observed in either group, the study was not powered to detect infrequent but clinically meaningful complications.

## Conclusion

The lattice tip catheter resulted in higher acute procedural success combined with reduced ablation and procedural time for radiofrequency ablation of the cavotricuspid isthmus compared to the standard irrigated focal-tip catheter. Future studies are needed to assess long-term efficacy and clinical outcomes.

## Data Availability

The data underlying this article will be shared on reasonable request to the corresponding author.
